# Diurnal patterns of gas-exchange and metabolic pools in tundra plants during three phases of the arctic growing season

**DOI:** 10.1002/ece3.467

**Published:** 2013-01-11

**Authors:** Rajit Patankar, Behzad Mortazavi, Steven F Oberbauer, Gregory Starr

**Affiliations:** 1 Department of Biological Sciences, University of AlabamaTuscaloosa, Alabama; 2 Dauphin Island Sea LaboratoryDauphin Island, Alabama; 3 Department of Biological Sciences, Florida International UniversityMiami, Florida

**Keywords:** Alaska, circadian clock, photoperiod, photosynthesis, respiration, sugars, total nonstructural carbohydrates, tussock tundra

## Abstract

Arctic tundra plant communities are subject to a short growing season that is the primary period in which carbon is sequestered for growth and survival. This period is often characterized by 24-h photoperiods for several months a year. To compensate for the short growing season tundra plants may extend their carbon uptake capacity on a diurnal basis, but whether this is true remains unknown. Here, we examined in situ diurnal patterns of physiological activity and foliar metabolites during the early, mid, and late growing season in seven arctic species under light-saturated conditions. We found clear diurnal patterns in photosynthesis and respiration, with midday peaks and midnight lulls indicative of circadian regulation. Diurnal patterns in foliar metabolite concentrations were less distinct between the species and across seasons, suggesting that metabolic pools are likely governed by proximate external factors. This understanding of diurnal physiology will also enhance the parameterization of process-based models, which will aid in better predicting future carbon dynamics for the tundra. This becomes even more critical considering the rapid changes that are occurring circumpolarly that are altering plant community structure, function, and ultimately regional and global carbon budgets.

## Introduction

Over half a century has passed since the initial studies on photosynthesis in arctic tundra vascular plants (Bliss 1956, 1962; Mooney and Billings 1961). However, surprisingly, given the unique, short growing season characterized by 24-h photoperiods, a fundamental question remains: Do arctic tundra plants show circadian rhythms in their photosynthetic cycles during the growing season? This lack of knowledge is all the more extraordinary given the recent trend toward understanding linkages in carbon dynamics across multiple scales (leaf to ecosystem) within the context of arctic climate warming (Chapin et al. 2005; McGuire et al. 2009; Post et al. 2009). To date, ecophysiological studies on arctic tundra plants have typically measured ambient or light-saturated midday values (e.g., Tieszen 1973; Johnson and Tieszen 1976; Defoliart et al. 1988; Oberbauer and Oechel 1989; Starr and Oberbauer 2003; Starr et al. 2008), which limits our understanding of arctic plant carbon acquisition to a narrow temporal window during the diurnal time course. Furthermore, whether tundra plants maintain or depart from predicted circadian rhythms would influence estimates of key ecosystem processes, such as primary productivity, carbon balance, and the parameterization of process-based ecosystem carbon models (such as GAS-FLUX, Tenhunen et al. 1994; SPA, Williams et al. 1996) that incorporate species-level physiological parameters. Thus, the need to understand diurnal patterns in arctic tundra vascular plants across diverse growth forms (evergreen, deciduous, graminoids) remains crucial for several compelling reasons.

A number of studies have revealed some generalized patterns in arctic plant carbon dynamics since Bliss's (1956) seminal work. Photosynthesis in arctic plants generally appear to have a nonlinear uni-modal relationship with temperature, with increases up to, and decreases after, a mean temperature of ∼15**°**C (tundra grasses, Tieszen^1973^; dwarf deciduous and evergreen species, Johnson and Tieszen 1976) indicating an inability to photosynthesize at peak rates beyond this range of moderate temperatures. Comparison of midday light-saturated photosynthesis between 19 vascular growth forms (Oberbauer and Oechel 1989) revealed that evergreens show the lowest photosynthetic rates compared to other growth forms (graminoids, forbes, and deciduous shrubs), a pattern consistent with prior area-based measurements of photosynthesis in arctic plants (e.g., Tieszen and Johnson 1975).

Seasonal patterns in midday plant physiology reveal that light-saturated photosynthesis is typically lowest right after melt out in the spring, peaks during the middle of the growing season (early July), and decreases approaching fall (Defoliart et al. 1988; Starr et al. 2000, 2008). Moreover, simulated warming and extended growing season do not appear to have measurable effects on photosynthetic rates in several species (Starr et al. 2000, 2008), suggesting adaptive constraints to physiological capacity. However, arctic sedges show reduced photosynthesis and stomatal conductance in response to reduced root temperatures, implying robust controls on gas-exchange rates by soil temperatures (Starr et al. 2004). Furthermore, long-term experimental manipulations of nutrients (nitrogen and phosphorus), air temperature, and light availability reveal that at the species level, photosynthesis responds most to increased light intensities (Chapin and Shaver 1996).

To date, only two prior studies have examined diurnal patterns from the Alaskan arctic tundra, but these have been limited a) in the number of species examined and b) to the peak of the growing season. Tieszen and Johnson (1975) reported on the diurnal pattern of photosynthetic uptake in the grass *Dupontia fischeri*, as part of a study on seasonal patterns of light-saturated photosynthesis in this species and found a peak in photosynthesis around midday, with the lowest uptake at night, corresponding with drops in temperature and reduced light intensity, (however, no formal methods were used to statistically test this relationship). Similar midday photosynthetic peaks were observed in two sedges (*Eriophorum vaginatum* and *E. angustifolium*) in moist and wet sites in the Alaskan arctic tundra, respectively, when measured under ambient light conditions (Gebauer et al. 1998). However, these midday peaks were observed on overcast days and peak photosynthetic rates in both species shifted to earlier in the day (between 7–8 am) with high light quantities and greater associated air temperatures. Both of these studies were confined to grasses and sedges, thereby leaving out other commonly occurring growth forms, such as deciduous and evergreen shrubs.

In arctic tundra plants, exposure to high light and low temperatures during spring melt out cause highly stressful conditions to the photosynthetic apparatus thus resulting in low photosynthetic efficiencies represented by low quantum yield values (Oberbauer and Starr 2002). Quantum yield of photosystem II measured as the ratio of variable to maximum chlorophyll fluorescence (Maxwell & Johnson 2000), is a common measure of plant “stress” associated with photosynthetic efficiency. Additionally, plants (insulated) under snow show visibly lower stress than those that are recently snow-free (Oberbauer et al. unpubl. data). However, with increasing soil and air temperatures, photosynthetic efficiencies increase rapidly to a maxima that remain fairly constant through much of the summer and into fall, when rates again drop as a result of leaf hardening and/or deteriorating leaf health (Oberbauer and Starr 2002).

Carbon metabolites produced as a direct result of photosynthesis are regulated by physical, photochemical, and biochemical component factors (Geiger and Servaites 1994) and as a result are likely to fluctuate based on diurnal differences in carbon sequestration. While prior studies have examined seasonal changes in metabolic carbon pools in diverse arctic tundra growth forms (Chapin et al. 1986; Olsrud and Christensen 2004), to our knowledge only one study has examined diurnal patterns of total foliar carbohydrates in *Oxyria digyna* (Warren Wilson 1954). This study found a diurnal pattern in total foliar carbohydrate concentrations during continuous “daylight”. Leaf total nonstructural carbohydrate (TNC) concentrations decreased or remained stable in deciduous shrubs, and increased in forbs, graminoids and evergreens as the growing season progresses toward fall (Chapin et al. 1986) implying an important role of TNCs as winter storage resources. High concentrations of TNCs found in stems and roots of deciduous species in the fall indicates that these plant components are the main storage organs for carbohydrate resource and are used for leaf growth in the spring. Thus, the varied growth forms found in the Arctic show distinct strategies in their TNC concentration toward survival and growth. However, when total sugars are considered in isolation, all growth forms show a consistent, uniform pattern with high foliar concentrations in the spring, significant drops in the summer and elevated concentrations in the fall (Chapin and Shaver 1988). However, the dynamics of diurnal gas-exchange and metabolite concentration patterns as the growing season progresses remain unknown.

Here, we examine diurnal patterns of leaf gas-exchange (photosynthesis and dark respiration), chlorophyll fluorescence, and metabolite concentrations in seven arctic tundra species during three distinct time periods (spring melt out, peak summer, and fall) of the short growing season. Furthermore, we determine which among a suite of environmental factors drive these patterns among the species and, finally we compare diurnally averaged patterns in leaf gas-exchange and metabolite concentration across the three time periods. On the basis of prior studies that found departures from circadian rhythms under constant high light conditions (Hennessey and Field 1991), we hypothesized that given continuous high-quality light, tundra plants would move away from circadian rhythms and photosynthesize at or close to, their (net) maximum capacity. Changes in photosynthetic assimilate concentrations (from neighboring plants) on the other hand were predicted to not change diurnally (Yang et al. 2002; Sun et al. 2009), but instead are influenced/governed by changing internal and external stressors such as temperature, water vapor deficits, or herbivory.

## Methods and Materials

### Study site and experimental design

The study was conducted in a moist acidic tussock tundra site at the Toolik Lake field station, Alaska (68°37′39″N, 149°35′51″W). Seven species representing four growth forms and comprising ∼80% of the vascular plant ground cover (Walker et al. 1994) were selected for the study. These included: *Vaccinium vitis-idaea* L., *Cassiope tetragona*, *Ledum palustra* L. (evergreen shrubs), *Betula nana* L., *Salix pulchra* Cham.(deciduous shrubs), *Eriophorum vaginatum* L.(wintergreen sedge), and *Carex bigelowii* Torr.(sedge). A detailed description of the community can be found in Bliss and Matveyeva (1992). To examine seasonal variations in diurnal ecophysiology, measurements were made: (1) immediately after melt out in the spring (30 May–7 June 2011), B) peak summer (8–20 July) and C) fall (7–17 September). Five of the seven species were measured immediately after melt out in May–June, *S. pulchra* and *C. bigelowii* were included from the July sampling period onward (as neither had fully developed foliage in the spring). We measured six individuals of each species at 4-h intervals (00:00, 04:00, 08:00, 12:00, 16:00, 20:00 hours) per 24-h sampling period.

### Diurnal physiological measurements

At each sampling interval, plants (*n* = 6 per species) were dark-adapted for a minimum of 10 min and *F*_*v*_*/F*_*m*_ (representing potential quantum efficiency of photosystem II [PSII]) was measured using an OS5-FL modulated-fluorometer (Opti-Sciences, Tyngsboro, Massachusetts). Immediately thereafter, *A*_*max*_ (maximum photosynthetic capacity at saturating light), stomatal conductance (*g*_*s*_) and net foliar dark respiration *R*_*d*_ were measured in situ at a constant reference CO_2_ concentration of 400 μmol m^−2^ s^−1^ using an infrared gas analyser (Li-6400XT portable photosynthesis system; LI-COR, Lincoln, Nebraska). *A*_*max*_ measurements were made at a saturating light intensity of 1500 μmol m^−2^ s^−1^ (Oberbauer and Oechel 1989; Starr et al. 2008; 1200 in the spring and fall), and following this, the chamber light source was switched off. *R*_*d*_ was measured after a period of ∼ 5–7 min in darkness, when dark respiratory rates had stabilized. Leaves were collected and brought back to the lab where leaf areas (Li-3100 area meter; LI-COR, Lincoln, Nebraska) were measured (to correct for area-based gas-exchange measurements) following the 24-h sampling period. Due to rain events and dew formation at several time intervals during the study period, leaves were often wet and despite best efforts, this lead to inaccurate/unreliable estimates of stomatal conductance. Hence diurnal patterns of field *g*_*s*_ measurements are not presented here. However, diurnally integrated values of *g*_*s*_ were used to examine correlations between *A*_*max*_ and *g*_*s*_ (Table 1).

**Table 1:** Correlation coefficients between Amax and gs in seven arctic tundra species from three time periods during the arctic growing season

	Melt out	Peak	Fall
*Betula nana*	0.011	0.146 *	0.013
*Salix pulchra*	–	0.514 ***	0.023
*Carex bigelowi*	–	0.474 ***	–
*Eriophorum vaginatum*	0.276**	0.745 ***	0.034
*Cassiope tetragona*	0.122*	0.006	0.067
*Vaccinium vitis-idea*	0.009	0.837 ***	0.156*
*Ledum palustre*	0.017	0.399 ***	0.178*

Level of significance (*P*) is represented by the “*” symbol as follows:

* *P* < 0.05.

** *P* < 0.01.

*** *P* < 0.001.

### Microclimate variables

Air (*T*_*air*_) and leaf temperature (*T*_*leaf*_), ambient pressure (*P*) and leaf to air vapor pressure deficit (*D*) were recorded along with gas-exchange parameters using the infrared gas analyser (Li-6400XT portable photosynthesis system; LI-COR, Lincoln Nebraska).

### Diurnal metabolite measurements

To examine diurnal differences in the partitioning of recently acquired carbon, foliar tissue from adjacent stems, as not to affect our physiological measurements, were collected separately at each time interval (*n* = 6 per time interval) and immediately stored in the field at −40°C and transferred within 30 min to a −80°C freezer. Samples were then freeze-dried for 24 h at −80°C (Labconco Co., Kansas City, Missouri), ground and extracted with 80% ethanol, and analyzed enzymatically for soluble sugars (glucose, fructose, sucrose) and starch concentrations according to Boehringer (1984).

### Statistical analyses

Data were examined for deviations from normality and in cases where this occurred, the data were normalized using appropriate transformations (Zar 1984). For each species, a one-way analysis of variance (ANOVA) was performed to determine differences in gas-exchange parameters and metabolite concentrations between the six diurnal time intervals. To test for differences in physiological parameters between seasons and species (and the interaction between them) diurnal data for the individual response variables were pooled as there was no significant variation in diurnal ranks between seasons and species (i.e., diurnals patterns were similar across seasons and species), and a two-way ANOVA was used with the above two variables as fixed factors (a student's *t*-test was used for *Salix pulchra* to determine seasonal differences between summer and fall; *C. bigelowi* was only measured during peak summer). To determine which environmental variables contributed to variations in physiological parameters, response (gas-exchange and metabolite) data from the different diurnal time intervals, and growing periods were pooled separately for each species, and a multiple linear regression model was run using three environmental variables – air temperature (**°**C), pressure (kPa), and leaf to air vapor pressure deficit *D*_L_ (kPa) – as predictors. Leaf temperature was highly correlated with air temperature, and was thus excluded from the analysis to avoid collinearity.

## Results

### Diurnal patterns of gas-exchange and chlorophyll fluorescence

**Figure 1:**
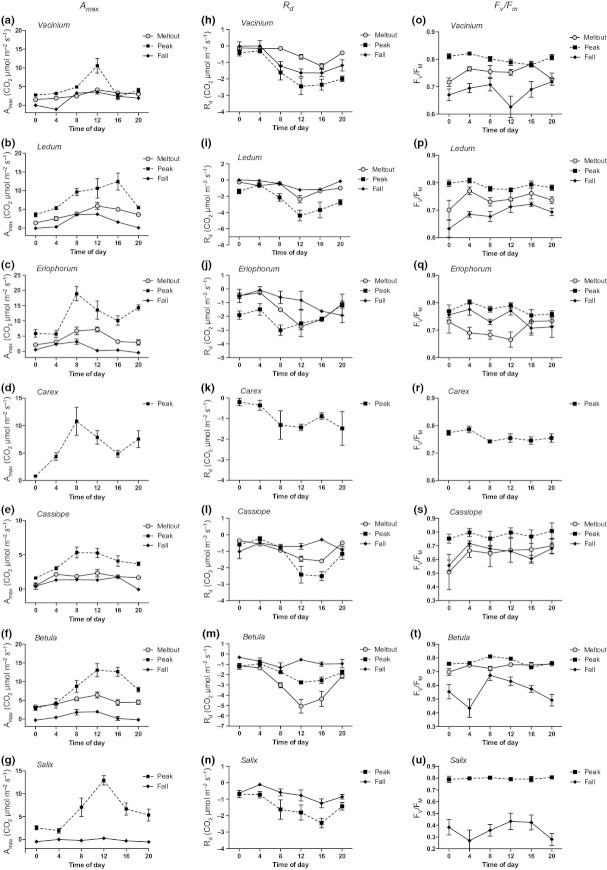
Diurnal patterns of gas exchange (light-saturated photosynthesis A_max_ [a–g], respiration [h–n]) and chlorophyll fluorescence (F_V_F_M_ [o–u]) from seven arctic tundra vascular plants from Toolik Lake Field Station, Alaska measured at three time periods (meltout, peak, fall) in 2010. Filled circles = melt out, filled squares = peak, and filled diamonds = fall period of the growing season. All symbols are means of *n* = 6 plants (+- 1 SEM); *x*-axis: diurnal time interval.

All five species examined following melt out showed peaks in *A*_*max*_ at midday (12:00 time interval) that were significantly higher than *A*_*max*_ measured at midnight (Fig. 1 a–g, Table 2). Leaf dark respiration similarly peaked at the 12:00 time interval in four species, but was highest at 16:00 in *Vaccinium* (Fig. 1 h–n). Diurnal differences in *F*_*v*_*/F*_*m*_ were only significant in *Cassiope* and this temporal variability was driven by the lower midnight value when compared to the other time intervals (Fig. 1 o–u).

**Table 2:** *F* and *P* values of analysis of variance (ANOVA)s examining diurnal patterns of gas exchange (photosynthesis and respiration) and chlorophyll fluorescence in seven arctic tundra vascular species

	A_max_			Respiration			*F*_*v*_*/F*_*m*_		
	Melt out	Peak	Fall	Melt out	Peak	Fall	Melt out	Peak	Fall
*B. nana*	3.73**	11.49***	4.22**	13.35***	7.41***	1.2	2.09	6.71***	3.36*
*S. pulchra*	–	10.39***	3.17*	–	3.43**	3.12*	–	1.68	1.13
*C. bigelowi*	–	6.26***	–	–	1.49	–	–	1.92	–
*E. vaginatum*	7.49***	7.99***	6.12***	31.75***	1.37	2.07	1.00	1.47	1.6
*C. tetragona*	4.87***	6.52***	9.16***	27.09***	8.27***	1.96	3.35**	1.81	4.37**
*V. vitis-idea*	8.68***	10.71***	22.57***	19.28***	8.35***	6.38***	2.29	2.81*	1.73
*L. palustre*	8.77***	5.47***	47.68***	14.41***	6.45***	11.2***	1.23	1.63	2.88*

Level of significance (*P*) is represented by the “*” symbol as follows:

* *P* < 0.05.

** *P* < 0.01.

*** *P* < 0.001. *B. nana = Betula nana*, *S. pulchra = Salix pulchra, C. bigelowi = Carex bigelowi, E. vaginatum = Eriophorum vaginatum, C. tetragona = Cassiope tetragona, V. vitis-idea = Vaccinium vitis-idea, L. palustre = Ledum palustre*.

Clear diurnal patterns were observed during peak summer (July), but unlike earlier in the season, peak time intervals in *A*_*max*_ differed between species. *A*_*max*_ in both grasses species (*Eriophorum* and *Carex*) peaked at 08:00 (Fig. 1 c, d) and at 12:00 in both deciduous species (*Betula* and *Salix*) (Fig. 1 f, g). Among the evergreens, *A*_*max*_ peaked at 12:00 in *Vaccinium,* at 16:00 *in Ledum* and reached similar levels at 08:00 and 12:00 in *Cassiope*. *R*_*d*_ was highest at 12:00 for *Vaccinium*, *Ledum*, and *Betula*, was highest at 08:00 for *Eriophorum*, 16:00 for *Salix* and peaked at 08:00, and 20:00 for *Cassiope* (Fig. 1 h–n). *F*_*v*_*/F*_*m*_ showed significant diurnal changes in only two of the seven species examined during the peak summer period (Table 2). In both species, these differences were driven by low values from a single time interval (16:00 in both *Betula* and *Vaccinium*) in comparison to other time intervals (Fig. 1 o, t).

*Carex* leaves had senesced before measurements commenced in the fall and were thus excluded from this time period. *A*_*max*_ in *Eriophorum* peaked at 08:00, and was significantly lower at 12:00 through midnight (Fig. 1 c). Among the evergreens, *Vaccinium* and *Ledum* both had *A*_*max*_ peaks at 08:00 and 12:00, whereas in *Cassiope,* rates were comparably high from 04:00 to 16:00 (Fig. 1 a, b, c). *A*_*max*_ in both *Betula* and *Salix* peaked at 12:00, and were significantly higher than at midnight (Fig. 1 f, g, Table 2). *R*_*d*_ rates during the fall showed diurnal peaks in five of six species examined, but differences between the time intervals were only significant in *Vaccinium*, *Ledum* and *Salix* (Table 2). *R*_*d*_ peaked at 12:00 in *Vaccinium* and *Ledum* and at 16:00 in *Salix* (Fig. 1 h, i, n). Diurnal differences in *F*_*v*_*/F*_*m*_ were significant in *Betula* and the evergreens *Cassiope* and *Ledum* (Table 2), and were driven by lower midnight values (*Cassiope, Ledum*) and 04:00 (*Betula*) compared to the other diurnal time intervals (Fig. 1 i, l, n).

### Diurnal patterns of metabolite pools

**Figure 2:**
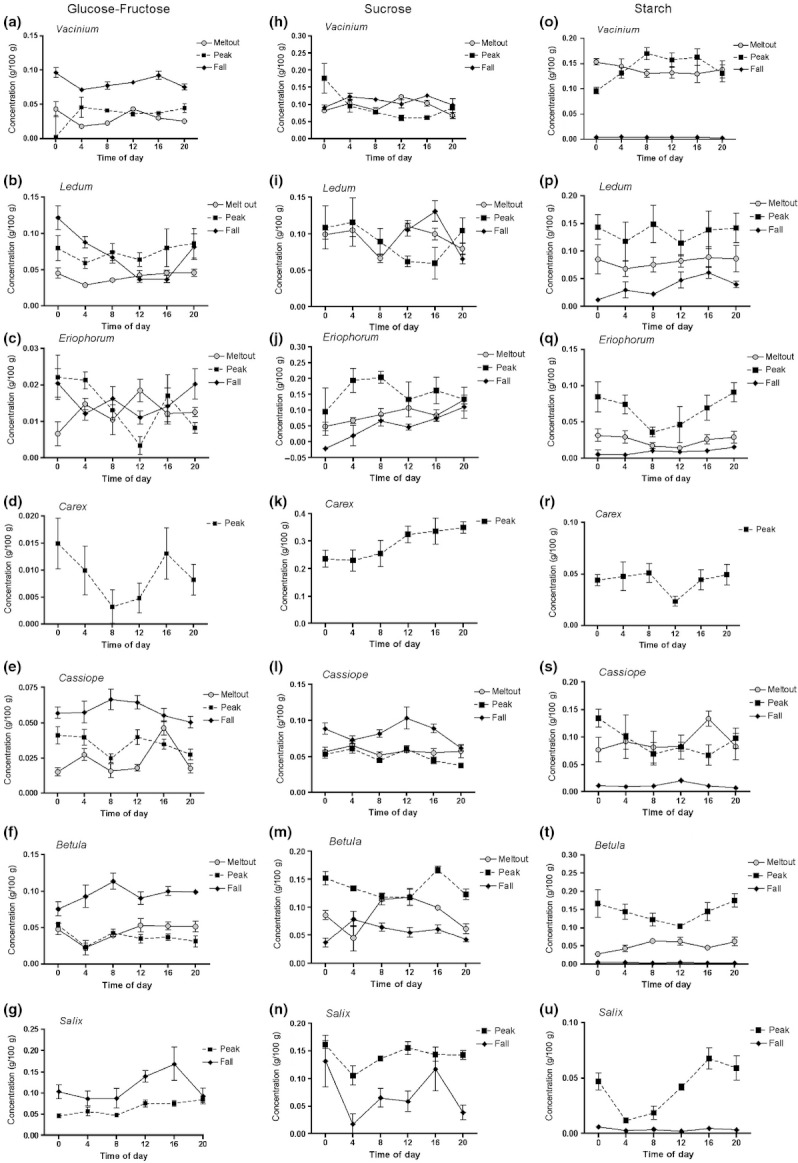
Diurnal patterns of concentration of metabolites (glucose-fructose [a–g], sucrose [h–n], starch [o–u]) from seven arctic tundra vascular plants from Toolik Lake Field Station, Alaska measured at three time periods (meltout, peak, fall) in 2010. Filled circles = melt out, filled squares = peak, and filled diamonds = fall period of the growing season. All symbols are means of *n* = 6 plants (+- 1 SEM); *x*-axis: diurnal time interval, *y*-axis = concentrations (grams per 100 grams of dry tissue mass).

Significant differences in diurnal patterns of metabolite concentration during the melt out period were seen in *Betula*, *Cassiope*, *Vaccinium*, and *Ledum* (Table 3). This was due to decreased glucose-fructose concentrations at time interval 0:400 in *Betula* and *Vaccinium*, and increased glucose-fructose concentrations at 12:00 in *Cassiope* (Fig. 2 a, e, f). Sucrose likewise varied in *Betula*, *Vaccinium*, and *Ledum* (Table 3), with concentrations significantly lower at 04:00 in *Betula*, at 08:00 in *Ledum*, and at 20:00 in *Vaccinium* compared to other times (Fig. 2 a, b, f). Starch concentration was significantly different between time intervals in *Betula* and *Cassiope*, with the main difference seen in low starch concentrations at 04:00 in *Betula* and significantly higher concentrations at 16:00 in *Cassiope* (Fig. 2 e, f).

**Table 3:** *F* and *P* values of analysis of variance (ANOVA)s examining diurnal patterns of metabolite concentration in seven arctic tundra vascular species

	Glucose-Fructose			Sucrose			Starch		
	Melt out	Peak	Fall	Melt out	Peak	Fall	Melt out	Peak	Fall
*B. nana*	2.90*	2.70*	1.65	5.79***	4.79**	3.06*	2.94*	1.62	0.63
*S. pulchra*	–	5.45**	2.10	–	3.26*	2.45	–	9.27***	8.92***
*C. bigelowi*	–	1.39	–	–	12.8***	–	–	1.18	–
*E. vaginatum*	2.27	3.67*	1.29	1.17	0.74	4.29**	1.18	1.63	1.82
*C. tetragona*	13.83***	4.89***	1.15	0.47	11.4***	6.69***	7.37***	12.1***	3.06*
*V. vitis-idea*	4.34**	0.54	3.66*	7.70***	3.99**	3.85**	0.59	4.17**	0.79
*L. palustre*	2.05	1.19	8.49***	5.22**	0.94	30.30***	1.06	1.10	2.92*

Level of significance (*P*) is represented by the “*” symbol as follows:

* *P* < 0.05,

** *P* < 0.01,

*** *P* < 0.001. *B. nana = Betula nana*, *S. pulchra = Salix pulchra, C. bigelowi = Carex bigelowi, E. vaginatum = Eriophorum vaginatum, C. tetragona = Cassiope tetragona, V. vitis-idea = Vaccinium vitis-idea, L. palustre = Ledum palustre*.

Six of the seven species examined during peak summer showed significant differences between time intervals in at least one of the three metabolites measured, *Ledum* being the exception. *Salix* and *Cassiope* showed diurnal differences in all three types of metabolites, whereas *Betula* and *Vaccinium* showed diurnal differences in two of the three measured metabolites (Table 3). The graminoids *Eriophorum* and *Carex* showed differences in glucose-fructose and sucrose, respectively (Table 3). Significant differences in *Salix* were driven by low glucose-fructose concentrations at midnight and at 08:00, low sucrose concentrations at 04:00, and low starch concentrations at 04:00 and 08:00 (Fig. 2 g, n, u). *Cassiop*e exhibited the opposite pattern, with significantly higher glucose-fructose, sucrose, and starch concentrations at midnight and 04:00 compared to other time intervals (Fig. 2e, l, s). Diurnal differences in *Betula* carbohydrate concentrations were driven by higher concentrations of glucose-fructose and sucrose at midnight and 16:00, respectively (Fig. 2 f, m), whereas in *Vaccinium*, differences were driven by high and low concentrations of sucrose and starch, respectively, at midnight (Fig. 2 a, o). Glucose-fructose concentration in *Eriophorum* was lowest at the 12:00 time interval (Fig. 2 c), whereas in *Carex* sucrose concentrations were lowest at midnight and at 04:00 (Fig. 2 d).

All six species measured in the fall showed diurnal differences in at least one of the three metabolites types. During this time period, *Ledum* had diurnal differences in the concentrations of all three metabolites (unlike in the summer where no diurnal differences were found in this species). Glucose-fructose concentrations in *Ledum* were significantly lower at 12:00 and 16:00, whereas sucrose and starch concentrations were significantly higher during these time intervals (Fig. 2 b, i, p). The other two evergreens, *Vaccinium* and *Cassiope* showed diurnal differences in glucose-fructose/sucrose and sucrose/starch concentrations, respectively. *Vaccinium* glucose-fructose concentrations were highest at midnight, whereas sucrose was highest at 16:00 (Fig. 2 a, h); *Cassiope* sucrose and starch concentrations were highest at noon (Fig. 2 l, s). Sucrose concentrations in *Eriophorum* and *Betula* were both significantly lower at midnight compared to other time intervals, whereas starch concentration in *Salix* was significantly higher at this time interval (Fig. 2 j, m, n).

### Seasonal differences in physiology and metabolite concentration

**Figure 3:**
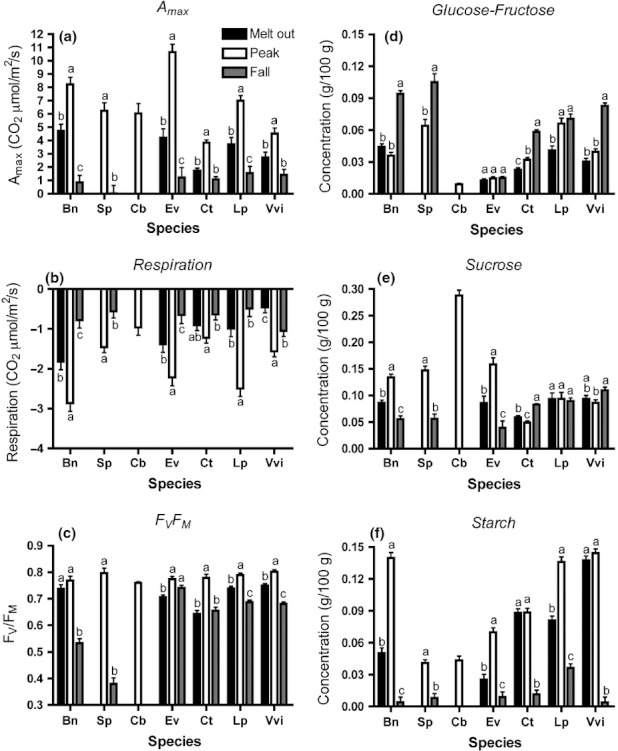
Seasonal differences in foliar gas-exchange, chlorophyll fluorescence and metabolite concentration in seven arctic tundra plants from Toolik Lake Field Station, Alaska in 2011. For each species, bars not connected by the same letters differ significantly at *P* < 0.05, and represent diurnally averaged means (+1 s.e.m). Black bars = melt out period (May–June), white bars = peak growing period (July), gray bars = fall (September).

Diurnally averaged *A*_*max*_ was significantly higher during the peak summer and lowest in the fall in all five species that were examined across these three time periods (*Salix* was not measured during melt out and *Carex* was only measured in the summer) (*F* = 41.94, *P* < 0.001, Fig. 3 a). *Cassiope* had the lowest melt out and summer *A*_*max*_ rates, whereas *Betula* and *Eriophorum* had the highest melt out and peak summer *A*_*max*_ values, respectively (Fig. 3a). Fall *A*_*max*_ rates did not vary significantly between species. *R*_*d*_ showed similar seasonal patterns as *A*_*max*_ except in the evergreen *Vaccinium* where *R*_*d*_ was lowest during melt out rather than fall (*F* = 15.55, *P* < 0.001; Fig. 3b). *Betula* had the highest spring and peak summer *R*_*d*_ rates, whereas *Vaccinium* and *Carex* had the lowest spring and summer rates, respectively (Fig. 3b). Similar to *A*_*max*_, fall *R*_*d*_ rates did not differ significantly between species. Chlorophyll fluorescence also peaked in the summer and were similarly high across all species (*F* = 38.52, *P* < 0.001; Fig. 3c). All species except *Eriophorum* showed significant declines in *F*_*v*_*/F*_*m*_ in the fall, with the deciduous species *Betula* and *Salix* exhibiting the largest drops (Fig. 3c). Fluorescence levels were similar in the five species examined during melt out (Fig. 3c). Glucose-fructose levels were highest in the fall across all species (*F* = 43.87, *P* < 0.001) except in the grass *Eriophorum*, which showed no seasonal differences. Glucose-fructose concentrations were consistently lowest during melt out except in *Betula*, where summer concentrations were marginally lower (*F* = 43.87, *P* < 0.001). The deciduous species had the highest fall concentrations of glucose-fructose, whereas the grasses *Carex* (summer) and *Eriophorum* (all periods) had the lowest concentrations among the seven species examined (Fig. 3d). Sucrose concentrations were highest in the peak summer among the deciduous (*Betula*, *Salix*) and graminoid (*Carex*, *Eriophorum*) species, but were either lower (*Cassiope*) or did not change (*Ledum*, *Vaccinium*) in the evergreens (*F* = 16.94, *P* < 0.001, Fig. 3e). Starch concentrations were consistently lowest in the fall in six of the seven species (*Carex* was excluded during this period) (*F* = 133.71, *P* < 0.001; Fig. 3f). Similarly, starch concentrations were highest in peak summer (significantly so in *Betula*, *Eriophorum*, and *Ledum*) (Fig. 3 f).

### Physiological and metabolic response to environmental variables

The linear multiple regression model revealed that (species-pooled) *A*_*max*_ and *R*_*d*_ were significantly correlated with (air) temperature (positive) and *D* (negative), but not with ambient pressure (*A*_*max*_: *r*^2^ = 0.373, *F* = 120.89, *P* < 0.001; *R*_*d*_: *r*^2^ = 0.391, *F* = 130.37, *P* < 0.001). *F*_*v*_*/F*_*m*_ was also positively correlated with temperature and negatively with *D*, although these correlations were weaker (*r*^2^ = 0.137, *F* = 32.19, *P* < 0.001). All three metabolite concentrations exhibited weak, but significant correlations with at least one, the environmental predictor. Glucose-fructose was weakly (negatively) correlated with temperature (*r*^2^ = 0.07, *F* = 15.39, *P* < 0.001), sucrose was correlated with temperature (positive) and pressure (negative) (*r*^2^ = 0.153, *F* = 36.73, *P* < 0.001), and starch was positively correlated with temperature and pressure (*r*^2^ = 0.162, *F* = 37.17, *P* < 0.001).

## Discussion

### Diurnal and seasonal patterns in leaf gas exchange

Contrary to our expectation, all seven species of arctic tundra vascular plants showed clear diurnal patterns in leaf gas-exchange with peak rates of photosynthesis and dark respiration around midday, and lulls at night. Arctic tundra plants thus appear to exhibit similar diurnal gas-exchange rhythms regardless of growth form (evergreens, graminoids or deciduous). The diurnal declines in carbon uptake during midnight and early morning hours persisted even under saturating light conditions during times when photoperiod was 24 h, suggesting that these species do not take advantage of light availability, but instead are constrained by a) stomatal water loss and b) internal physiological adaptations governed by circadian clocks (Geiger and Servaites 1994; Dodd et al. 2005). While diurnal patterns of stomatal conductance were not consistent across species (data not shown), daily averaged conductance values were strongly correlated with daily photosynthesis values, at least during the peak summer (Table 1), suggesting strong controls of water loss on leaf carbon uptake. Our hypothesis was based on prior lab and greenhouse studies on common species that showed departures from photosynthetic circadian rhythms under constant, uniform light conditions. For example, Hennessey and Field (1991) examined red kidney bean (*Phaseolus vulgaris* L.) photosynthetic circadian rhythms under tightly controlled greenhouse conditions and found that clear circadian rhythms were maintained at constant air temperatures and ambient CO_2_ concentrations under a fixed light-dark photoperiod, but did not persist under a constant low light regime (200 μmol m^−2^ s^−1^). There are compelling alternative explanations for the observed diurnal patterns. First, in our study, while plants were measured at a constant PPFD (just above saturation point) and a fixed CO_2_ concentration, the plants were not continuously sampled and thus no individual leaf or tiller was subjected to long periods of constant light. Hence it is probable that extended periods of high light exposure (and not “spot” measurements) on individual leaves might alter the observed diurnal patterns. Alternatively, plants in the field experience far from “ideal” environments, and are thus subject to a number of stresses (temperature, light, water status, nutrient availability, herbivory) that act in a mutually nonexclusive manner, and are thus likely to maintain circadian patterns to optimize the costs and benefits of carbon uptake (Dodd et al. 2005). Additionally, the plants in this study experienced high levels of light intensities during peak summer (peak summer midday PAR was frequently above 1000 μmol m^−2^ s^−1^) and were measured at PAR levels well above saturation. Hence, we suggest, based on this and prior studies measuring in situ diurnal patterns in the arctic (Tieszen 1973; Gebauer et al. 1998), that arctic plants are constrained in their ability to fully exploit available light, and this is likely due to a combination of adaptive constraints, such as stomatal water loss, and diurnal circadian clocks (which might in fact confer significant advantages to photosynthesis, likely leading to greater growth and survival; see Dodd et al. 2005 for a recent example). This is further reinforced by the fact that while prior studies examining arctic plants (Tieszen 1973; Gebauer et al. 1998) have shown a close matching between diurnal photosynthesis and photoperiod (implying external regulation/influence), we show that departures from normal diurnal light regimes (i.e., artificially provisioned saturating light levels) do not result in modifications to diurnal patterns (implying internal control). Any measured differences in PS II efficiency, on the other hand, had more to do with large changes between any two times intervals, as opposed to diurnal changes consistent with gas-exchange parameters implying, for the most part, minimal diurnal variation in the photochemical pathway of host leaves.

While marked diurnal patterns in gas exchange were observed, the timing of peaks and lulls differed between species. Photosynthetic capacity in both graminoids (*Eriophorum* and *Carex*) peaked relatively soon after lulls in midnight and early morning intervals, as seen by the peaks at ∼08:00 during summer in both species, whereas peaks were closer to noon or even 16:00 in the evergreens and deciduous species. Corresponding respiration rates in the two graminoids peaked at similar times, as opposed to the lags in peak respiration seen in three of the five other species (diurnal respiration rates in *Carex*, however, were not significant). Similar early morning photosynthetic peaks were observed in *Eriophorum vaginatum* and *E. augustifolium* measured at ambient light conditions on a sunny day in arctic tussock tundra (Gebauer et al. 1998), but shifted toward midday under overcast conditions. This shift was attributed to photosynthetic responses of tillers to water stress, and more specifically external (leaf to air vapor deficit) rather than internal (water potential) water stress. Given the lack of a significant association between *A*_*max*_ (and also respiration) and *D*_*L*_ in both graminoids (data not shown), our findings suggest that irrespective of season, peak diurnal *A*_*max*_ rates in the graminoids are reached relatively early during the day thereby suggesting a requirement to accelerate carbon acquisition as early as possible. Diurnally averaged *A*_*max*_ showed a consistent seasonal pattern, with the highest rates in July (peak) and the lowest rates in the September (fall). Senescing leaves, largely devoid/depleted of chlorophyll, likely resulted in the low rates of *A*_*max*_ (∼ 0–1 μmol m^−2^ s^−1^) seen in the deciduous species *Betula* and *Salix* in the fall. Midday respiration rates were highest during the peak summer, except in *Betula*, where spring rates were noticeably higher. As mentioned previously, diurnal peaks in respiration lagged behind peak *A*_*max*_ rates in *Vaccinium*, *Cassiope* and *Salix*, suggesting a recovery phase after potentially significant water loss in these species. Diurnally averaged seasonal trends here resembled those of *A*_*max*_, with high midsummer rates and the lowest respiration rates in the fall, (except in *V. vitis-idea* where spring rates were lowest). Dark-adapted PS II quantum efficiency rates generally remained relatively constant during a 24-hr period, suggesting that PS II quantum efficiency does not fluctuate diurnally, but rather changes as the growing season progresses, as seen by the peak values in July and declines in fall (Fig. 3). Observed seasonal trends in all three physiological traits are consistent with prior studies that have examined seasonal changes in leaf physiology in arctic tundra plants, with midsummer peaks in *A*_*max*_, *R*_*d*_, and *F*_*v*_*/F*_*m*_ (see Defoliart et al. 1988; Starr et al. 2008). The significant decreases in fall gas-exchange traits compared to the other two seasons is likely due to declines in leaf functioning in response to decreases in diurnal photoperiods from a summer peak of 24 h to about ∼14 h in the fall.

### Diurnal and seasonal patterns in leaf metabolites

Unlike with leaf physiological traits, diurnal patterns in leaf metabolic pools were less consistent, with patterns changing across seasons and growth forms (Fig. 2). In many instances, no clear diurnal patterns were detected, as we had predicted based on earlier findings (Yang et al. 2002; Sun et al. 2009). Glucose-fructose concentrations were generally lowest between ∼08:00 and noon in most species; however, there were no significant diurnal changes in glucose-fructose concentrations in at least one time period for all the species, and several of the patterns were marginally significant (e.g., melt out and peak values for *Betula*, peak values for *Eriophorum*, and fall values for *Vaccinium*). Decreased concentrations of reducing sugars around midday could imply depletion on account of high respiration rates, but no strong correlations were detected between respiration and glucose-fructose concentrations for any of the species (data not shown). All species displayed diurnal changes in sucrose concentrations from at least one time during the growing season, but patterns across growth forms are not entirely consistent. The evergreens *Vaccinium* and *Ledum* showed peaks in sucrose concentrations between noon and 16:00 during the spring and fall, and the reverse (lulls) at this time intervals during the peak growing season, which likely corresponds with increased respiration rates during these time intervals. Diurnal patterns of sucrose concentrations in *Cassiope,* on the other hand, showed significant increases during the midday time interval during peak and fall time periods. Both graminoids had the highest concentrations at 20:00 (*Eriophorum* – fall, *Carex* – peak) and the lowest at midnight (*Eriophorum* – all periods, *Carex* – peak). Patterns of sucrose concentration were highly variable among the deciduous shrubs, especially in the fall. Interestingly in all but the fall *Betula* diurnal, midnight concentrations were amongst the highest followed by marked declines at the 04:00 interval, suggesting possible increases in sucrose consumption toward physiological processes immediately following midnight lulls. Strong diurnal patterns in starch concentration were confined to a couple of time periods from only three (of seven) species. Among the evergreens, *Vaccinium* had elevated midday starch concentrations during the peak season, whereas in *Cassiope*, the same pattern was seen during the melt out and fall seasons (this trend was opposite during the peak growing season). *Salix* summer starch concentrations appeared lowest during the early daytime hours before steadily increasing to a peak at 16:00. Overall, our findings point to some diurnal trends in the carbohydrates, but no consistent patterns based on plant growth forms. This is not entirely unexpected as changes in fast turnover metabolites are likely to be highly sensitive to fluctuations in a combination of internal functional (stomatal regulation, carbon uptake, biochemical pathways) and external (climatic, edaphic) factors, thus resulting in varied patterns even over a diurnal time course. Starch concentrations were mostly not sensitive to diurnal changes, similar to other studies that have examined foliar starch concentrations in the field (Green et al. 2002).

Diurnally averaged glucose-fructose concentrations were highest in the fall in all species except in the graminoid *Eriophorum*, which do not alter concentrations across the growing season (Fig. 3d). This species also had the lowest concentrations amongst all the species that were examined from at least two time periods in the summer. Increases in reducing sugar (glucose + fructose) concentrations in the fall could be a consequence of declines in carbon demands wherein fast turnover sugars are not consumed as rapidly as during melt out and peak summer periods, although this does not explain the higher fall concentrations measured in senescing deciduous leaves that are about to be shed. A more likely explanation for that latter observation is that senescing deciduous leaves often contain elevated levels of reducing sugars, which are thought to contribute toward accelerated senescence (Wingler et al. 2004, 2006; Yuanyuan et al. 2009). In the overwintering species, reducing sugars in leaves also likely play a role in freezing resistance enabling leaf survival through the winter (Levitt 1980). Distinct seasonal differences in diurnally averaged sucrose concentrations were measured between the various growth forms: in essence sucrose concentrations were highest during the peak summer period among the deciduous shrubs and graminoids, and highest in the fall among the evergreens (Fig. 3e). Like glucose/fructose, sucrose could also play a vital role in freeze tolerance among evergreen leaves, thus explaining the higher levels seen in the fall in these species. This result is similar to prior examinations on carbohydrate content in arctic plants, where increases in fall sugar concentrations have been observed across a number of growth forms (Chapin and Shaver 1988). A striking commonality between all the study species was the marked decline in foliar starch concentrations in the fall compared with melt out and peak summer levels (Fig. 3f). Conversion of starch into glucose (to aid in senescence (deciduous) or cold tolerance (evergreens)) or translocation to belowground parts as winter approaches is a likely reason for severely depleted foliage starch concentrations in fall. While seasonal patterns of TNC concentrations in tundra plants have been examined before (Chapin et al. 1986) this study is the first highlight changes in constituent carbohydrate fractions of TNC diurnally across the growing season.

Species-pooled gas-exchange variables showed typical responses to the environmental factors with *A*_*max*_ and *R*_*d*_ both responding positively to increases in temperature and negatively to *D*, although at very high temperatures photo-inhibitory effects will likely alter the linear nature of *A*_*max*_-temperature relationships. The weak, variable relationships seen between the carbohydrates and environmental variables might be the result of pooling foliage from neighboring plants in addition to the highly heterogeneous micro-topographical and climatic conditions that potentially influence carbon concentration in leaves.

Arctic tundra plants are subject to a number of dynamic environmental variables within the time course of a day. Large changes in light, temperature, precipitation (including snow), air, and soil moisture can be observed within relatively short spans (in hours), which in turn has the potential to influence the physiological functioning of plants. We show for the first time that vascular tundra plants from three distinct growth forms maintain clear diurnal patterns in carbon gas-exchange (photosynthesis and respiration) across three distinct periods of the growing season: (1) spring melt out, a period of increasing photoperiod, high light radiation and low temperatures (Starr and Oberbauer 2003); (2) the summer, which is typically characterized by long photoperiods and warmer air temperatures; and (3) in the fall, when photoperiod is greatly reduced and nighttime temperatures occasionally drop below freezing. While marginal shifts in gas-exchange peaks can be attributed to external factors (*viz* Gebauer et al. 1998), the observed diurnal patterns appear to be governed to a degree by internal circadian rhythms irrespective of seasonal differences in the environment (diurnal patterns of carbon uptake were similar even though correlations with water loss varied between peak summer and the fringe periods). The diurnal concentration of key metabolites is more varied, with reducing sugars and sucrose levels peaking at different times of the day in different species. The fate of these fast turnover sugars is thus more likely governed by the immediate responses by plants to shifts in external stressors than tied to strict internal circadian control. Finally, seasonal differences in foliar carbon metabolite concentrations reveal patterns in the different growth forms.

The arctic tundra today is experiencing unprecedented ecosystem-wide changes including substantial alterations in ecosystem carbon balance (The Intergovernmental Panel on Climate Change (IPCC) 2007; McGuire et al. 2009; Schuur et al. 2009), increase in the growing season length (Tedesco et al. 2009), warmer summers (Chapin et al. 2005) and winters (Bokhorst et al. 2009) and shifts in plant community composition (Sturm et al. 2001; Myers-Smith et al. 2011). In order to better understand the contribution of local vegetation to regional carbon cycles, several studies in the past have examined photosynthetic capacities of arctic vascular plants at various times in the season and in different habitats (Defoliart et al. 1988; Chapin and Shaver 1996; Starr et al. 2000, 2008). However, barring a single study on two tundra graminoids (Gebauer et al. 1998), there have been no prior explicit examinations on the diurnal functioning of arctic plants, and none across the growing season; with almost all prior studies have focussed on midday measurements of plant carbon gain and loss. Relying on midday and mid season values of net photosynthetic rates is likely to result in an overestimation of actual diurnally and seasonally integrated values. For example, diurnally averaged *A*_*max*_ is significantly lower and is ∼80% (3.4 vs. 4.3 μmol m^−2^ s^−1^), ∼75% (6.9 vs. 9.2 μmol m^−2^ s^−1^) and ∼ 58% (0.89 vs. 1.6 μmol m^−2^ s^−1^) that of peak midday values in the spring, summer, and fall, respectively (from pooled data of five species examined during all three seasons), implying nontrivial differences in net carbon uptake. More strikingly, diurnally averaged *A*_*max*_ from these five species were only ∼50% of peak summer rates in the spring and ∼13% of summer rates in the fall. Thus, the need to incorporate diurnally and seasonally integrated values of photosynthesis will substantially strengthen ecosystem-level studies and process-based models (e.g., SPA and GAS-FLUX) predictions of future carbon dynamics. Additionally, our findings on diurnal and seasonal foliar metabolite use/concentration shed light on a previously undocumented aspect of arctic plant physiology, and help explain how growth forms differ in their survival during and after the peak growing season. These finding become even more critical considering the rapid changes that are occurring circumpolarly that are altering plant community structure, function and ultimately region and global carbon budgets.

## References

[b1] Bliss LC (1956). A comparison of plant development in arctic and alpine tundras. Ecol. Monogr.

[b2] Bliss LC (1962). Adaptations of arctic and alpine plants to environmental conditions. Arctic.

[b3] Bliss LC, Matveyeva NV, Chapin FS, Jeffreis RL, Reynolds JF, Shaver GR, Svoboda J (1992). Circumpolararctic vegetation. Arctic ecosystems in a changing climate: an ecophysiological perspective.

[b4] Boehringer SA (1984). Methods of enzymatic food analysis using single reagents.

[b5] Bokhorst SF, Bjerke JW, Tømmervik H, Callaghan TV, Phoenix GK (2009). Winter warming events damage sub-Arctic vegetation: consistent evidence from an experimental manipulation and a natural event. J. Ecol.

[b6] Chapin FS, Shaver G (1988). Differences in carbon and nutrient fractions among arctic growth forms. Oecologia.

[b7] Chapin FS, Shaver G (1996). Physiological and growth responses of arctic plants to a field experiment simulating climatic change. Ecology.

[b8] Chapin FS, Mckendrik J, Johnson D (1986). Seasonal changes in carbon fractions in Alaskan tundra plants of differing growth form – Implications for herbivory. J. Ecol.

[b9] Chapin FS, Sturm M, Serreze MC, McFadden JP, Key JR, Lloyd AH (2005). Role of land-surface changes in arctic summer warming. Science.

[b10] Defoliart L, Griffith M, Chapin FS, Jonasson S (1988). Seasonal patterns of photosynthesis and nutrient storage in *Eriophorum vaginatum* L., an arctic sedge. Funct. Ecol.

[b11] Dodd AN, Salathia N, Hall A, Kévei E, Tóth R, Nagy F (2005). Plant circadian clocks increase photosynthesis, growth, survival, and competitive advantage. Science.

[b12] Gebauer R, Reynolds LE, Tenhunen JD (1998). Diurnal patterns of CO_2_ and H_2_O exchange of the Arctic sedges *Eriophorum angustifolium* and *E. vaginatum* (Cyperaceae). Am. J. Bot.

[b100] Geiger DR, Servaites JC (1994). Diurnal regulation of carbon in C3 plants. Ann. Rev. Plant Biol.

[b13] Green RM, Tingay S, Wang Z-Y, Tobin EM (2002). Circadian rhythms confer a higher level of fitness to *Arabidopsis* plants. Plant Physiol.

[b14] Hennessey TL, Field CB (1991). Circadian rhythms in photosynthesis: oscillations in carbon assimilation and stomatal conductance under constant conditions. Plant Physiol.

[b15] Johnson DA, Tieszen LL (1976). Aboveground biomass allocation, leaf growth, and photosynthesis patterns in tundra plant forms in Arctic Alaska. Oecologia.

[b16] Levitt J (1980). Responses of plants to environmental stress.

[b101] Maxwell K, Johnson GN (2000). Chlorophyll fluorescence - a practical guide. J. Exp. Bot.

[b17] McGuire AD, Anderson LG, Christensen TR, Dallimore S, Guo L, Hayes DJ (2009). Sensitivity of the carbon cycle in the Arctic to climate change. Ecol. Monogr.

[b18] Mooney HA, Billings WD (1961). Comparative physiological ecology of arctic and alpine populations of *Oryria digyna*. Ecol. Monogr.

[b19] Myers-Smith IH, Hik DS, Kennedy C, Cooley D, Johnstone JF, Kenney AJ (2011). Expansion of canopy-forming willows over the twentieth century on Herschel Island, Yukon Territory, Canada. Ambio.

[b20] Oberbauer SF, Oechel WC (1989). Maximum CO_2-_ assimilation rates of vascular plants on an Alaskan arctic tundra slope. Ecography.

[b21] Oberbauer SF, Starr G (2002). The role of anthocyanins for photosynthesis of Alaskan arctic evergreens during snowmelt. Adv. Bot. Res.

[b22] Olsrud M, Christensen TR (2004). Carbon cycling in subarctic tundra; seasonal variation in ecosystem partitioning based on in situ ^14^C pulse-labelling. Soil Biol. Biochem.

[b23] Post E, Forchhammer MC, Bret-Harte MS, Callaghan TV, Christensen TR, Elberling B (2009). Ecological dynamics across the Arctic associated with recent climate change. Science.

[b24] Schuur EA, Bockheim J, Canadell JG, Euskirchen E, Field CB, Goryachkin SV (2009). Vulnerability of permafrost carbon to climate change: implications for the global carbon cycle. Bioscience.

[b25] Starr G, Oberbauer SF (2003). Photosynthesis of arctic evergreens under snow: implications for tundra ecosystem carbon balance. Ecology.

[b26] Starr G, Oberbauer SF, Pop E (2000). Effects of lengthened growing season and soil warming on the phenology and physiology of *Polygonum bistorta*. Glob. Change Biol.

[b27] Starr G, Neuman DS, Oberbauer SF (2004). Ecophysiological analysis of two arctic sedges under reduced root temperatures. Physiol. Plant.

[b28] Starr G, Oberbauer SF, Ahlquist LE (2008). The photosynthetic response of Alaskan tundra plants to increased season length and soil warming. Arct. Antarct. Alp. Res.

[b29] Sturm M, Racine C, Tape K (2001). Climate change: increasing shrub abundance in the Arctic. Nature.

[b30] Sun W, Resco V, Williams DG (2009). Diurnal and seasonal variation in the carbon isotope composition of leaf dark-respired CO_2_ in velvet mesquite (*Prosopis velutina*. Plant Cell Environ.

[b31] Tedesco M, Brodzik M, Armstrong R, Savoie M, Ramage J (2009). Pan arctic terrestrial snowmelt trends (1979–2008) from spaceborne passive microwave data and correlation with the arctic oscillation. Geophys. Res. Lett.

[b32] Tenhunen JD, Siegwolf RA, Oberbauer SF, Schulze E-D, Caldwell MM (1994). Effects of phenology, physiology and gradients in community composition structure and microclimate on tundra ecosystem CO_2_ exchange. Ecological studies, vol. 100: ecophysiology of photosynthesis.

[b33] The Intergovernmental Panel on Climate Change (IPCC) Change (2007). Contribution of Working Group I to the Fourth Assessment Report of the IPCC. The physical science basis.

[b34] Tieszen LL (1973). Photosynthesis and respiration in arctic tundra grasses: field light intensity and temperature responses. Arct. Alp. Res.

[b35] Tieszen LL, Johnson DA (1975). Seasonal pattern of photosynthesis in individual grass leaves and other plant parts in arctic Alaska with a portable ^14^CO_2_ system. Bot. Gaz.

[b36] Walker MD, Walker DA, Auerbach NA (1994). Plant-communities of a tussock tundra landscape in the Brooks Range foothills, Alaska. J. Veg. Sci.

[b37] Warren Wilson J (1954). The influence of “midnight sun” conditions on certain rhythms in *Oxyria digyna*. J. Ecol.

[b102] Williams M, Rastetter ER, Fernandes DN, Goulden ML, Wofsy SC, Shaver GR (1996). Modelling the soil-plant-atmosphere continuum in a Quercus-Acer stand at Harvard Forest: the regulation of stomatal conductance by light, nitrogen and soil/plant hydraulic properties. Plant Cell Environ.

[b38] Wingler A, Mares M, Pourtau N (2004). Spatial patterns and metabolic regulation of photosynthetic parameters during leaf senescence. New Phytol.

[b39] Wingler A, Purdy S, MacLean JA, Pourtau N (2006). The role of sugars in integrating environmental signals during the regulation of leaf senescence. J. Exp. Bot.

[b40] Yang WQ, Murthy R, King P, Topa MA (2002). Diurnal changes in gas exchange and carbon partitioning in needles of fast-and slow-growing families of loblolly pine (*Pinus taeda*. Tree Physiol.

[b41] Yuanyuan M, Yali Z, Jiang L, Hongbo S (2009). Roles of plant soluble sugars and their responses to plant cold stress. Afr. J. Biotechnol.

[b42] Zar JH (1984). Biostatistical analyses.

